# Supplementary material for: A Likelihood Approach for Real-Time Calibration of Stochastic Compartmental Epidemic Models
Source: PLoS Comput Biol. 2017 Jan 17;13(1):e1005257. doi: 10.1371/journal.pcbi.1005257 (PMC5240920; doi:10.1371/journal.pcbi.1005257)
Supplement: S1 File — (TAR.GZ) [file pcbi.1005257.s014.tar.gz › HSPH_Online-SI-Revision/MSS10/n100-extreme/n100-extreme_NEW_table0.pdf]

1	R0	{2.76052, 16.3913, 0.1}	{2.48103, 13.7658, 2.}	{{2.48103, 13.7658, 2.}, {2.48103, 13.7658, 2.}}	{10.1246, 16.0179, 1900.}	inf	26 580.	22 969.9	{22 834.5, 23 090.7}	13.5821	0.0634012	newly	10 855.	10 994.2	{10 888., 11 101.}	1.30309	0.00561158	Reff	0.831027	0.836458	{0.832633, 0.839744}	0.658007	ar	92 090.	89 223.5	{89 059., 89 428.}	3.11268
2	R0	{2.18202, 15.1734, 3.}	{2.23539, 16.5205, 3.}	{{2.24606, 16.668, 3.}, {2.24606, 16.668, 3.}}	{3.13093, 9.83723, 0.}	inf	17 999.	18 808.6	{18 838.6, 19 092.3}	5.83115	0.0249444	newly	8027.	7671.96	{7559., 7786.}	4.42307	0.019674	Reff	0.873157	0.876071	{0.873985, 0.879356}	0.462394	ar	84 264.	84 974.	{84 850., 85 425.}	1.24867
3	R0	{2.57891, 12.5971, 1.}	{2.38758, 11.1331, 1.}	{{2.38758, 11.1331, 1.}, {2.38758, 11.1331, 1.}}	{7.41921, 11.6216, 0.}	inf	22 711.	19 848.6	{19 704., 19 968.1}	12.6037	0.0585135	newly	11 421.	11 284.7	{11 153., 11 398.}	1.26232	0.00552888	Reff	0.727149	0.741543	{0.738314, 0.744824}	1.97951	ar	90 302.	87 610.4	{87 417., 87 790.}	2.98066
4	R0	{2.5856, 9.28508, 0.1}	{2.61425, 9.30519, 3.}	{{2.21381, 7.10188, 3.}, {2.81311, 10.4354, 3.}}	{8.87297, 12.9135, 2900.}	inf	23 144.	23 430.2	{17 477.2, 26 060.1}	14.2806	0.0644299	newly	15 345.	15 476.3	{14 846., 15 801.}	2.41212	0.0104431	Reff	0.760942	0.75314	{0.740835, 0.776847}	2.04335	ar	90 312.	90 133.1	{84 596., 92 479.}	3.17729
5	R0	{2.4406, 12.1341, 1.}	{2.03104, 8.56733, 1.23}	{{2.03307, 8.59564, 1.}, {2.06386, 8.81239, 3.}}	{16.7809, 29.3946, 25.}	inf	22 895.	15 724.1	{15 568.8, 16 213.}	31.3209	0.163809	newly	12 782.	12 219.7	{12 028., 12 404.}	4.39916	0.0195779	Reff	0.972189	0.954378	{0.951111, 0.958641}	1.83196	ar	88 597.	80 386.9	{80 146., 80 987.}	9.26678
6	R0	{2.72155, 15.4094, 2.}	{2.69382, 15.1192, 0.76}	{{1.93419, 8.56054, 0.}, {2.93371, 17.1903, 1.}}	{12.8679, 19.4505, 62.}	inf	26 147.	25 848.5	{13 373.4, 29 900.3}	22.2113	0.112323	newly	11 271.	11 198.5	{10 161., 11 614.}	4.01588	0.0178815	Reff	0.880857	0.850125	{0.839213, 0.872584}	3.48892	ar	91 682.	89 915.7	{77 539., 93 919.}	5.46792
7	R0	{2.63781, 14.3935, 0.1}	{1.83586, 7.22097, 5.}	{{1.83586, 7.22097, 5.}, {1.83586, 7.22097, 5.}}	{30.4021, 49.8317, 4900.}	inf	24 939.	11 162.1	{11 005.4, 11 343.4}	55.2424	0.349167	newly	11 237.	9634.63	{9461., 9792.}	14.2598	0.0668553	Reff	0.790499	0.803096	{0.799724, 0.805907}	1.59351	ar	91 192.	74 524.	{74 134., 74 883.}	18.2779
8	R0	{2.82391, 16.104, 1.}	{2.93371, 17.1903, 1.}	{{2.93371, 17.1903, 1.}, {2.93371, 17.1903, 1.}}	{3.88808, 6.74551, 0.}	inf	27 368.	28 730.	{28 576.4, 28 871.5}	4.9766	0.0210887	newly	11 207.	11 152.9	{11 008., 11 281.}	0.779959	0.00340324	Reff	0.861801	0.855351	{0.851045, 0.859724}	0.762856	ar	92 664.	93 546.7	{93 416., 93 684.}	0.952625
9	R0	{2.80645, 14.6457, 0.1}	{2.02443, 8.5137, 0.98}	{{2.03307, 8.59564, 1.}, {2.03307, 8.59564, 1.}}	{27.8652, 41.8689, 884.}	inf	27 606.	15 603.4	{15 520.4, 15 942.2}	43.4782	0.248803	newly	12 694.	12 081.3	{11 907., 12 255.}	4.82693	0.0215467	Reff	0.94945	0.927411	{0.924283, 0.932815}	2.32123	ar	92 567.	80 253.3	{80 174., 80 806.}	13.3025
10	R0	{1.86213, 5.4271, 0.1}	{1.95989, 6.0959, 1.24}	{{1.90341, 5.70177, 0.}, {1.9862, 6.28417, 4.}}	{5.24964, 12.3234, 1278.}	inf	10 896.	12 897.	{11 710.6, 13 612.4}	18.3642	0.0724128	newly	11 765.	12 514.1	{12 025., 12 857.}	6.36753	0.0266671	Reff	0.763157	0.763718	{0.758035, 0.767852}	0.358746	ar	75 362.	78 515.6	{76 587., 79 610.}	4.18454
11	R0	{2.27349, 14.6338, 1.}	{1.88056, 10.2217, 3.}	{{1.88056, 10.2217, 3.}, {1.88056, 10.2217, 3.}}	{17.2833, 30.1503, 200.}	inf	19 721.	12 282.5	{12 126.4, 12 420.7}	37.7186	0.205661	newly	8893.	7877.62	{7739., 7988.}	11.4177	0.052684	Reff	0.854264	0.846504	{0.843834, 0.84927}	0.908378	ar	85 805.	75 871.6	{75 412., 76 232.}	11.5767
12	R0	{2.77261, 14.1894, 0.1}	{1.75316, 5.91037, 0.95}	{{1.75359, 5.92488, 1.}, {1.75359, 5.92488, 1.}}	{36.7687, 58.3466, 860.}	inf	27 138.	10 194.	{9937.28, 10 395.3}	62.4365	0.425294	newly	12 745.	10 695.	{10 427., 10 915.}	16.085	0.0762173	Reff	0.865526	0.844336	{0.840912, 0.847677}	2.44815	ar	92 508.	71 431.7	{71 048., 71 916.}	22.7832
13	R0	{2.65402, 19.3935, 2.}	{1.87584, 10.2502, 2.}	{{1.87584, 10.2502, 2.}, {1.87584, 10.2502, 2.}}	{29.3206, 47.1461, 0.}	inf	25 765.	13 177.9	{12 998.7, 13 344.4}	48.8536	0.291205	newly	8948.	8643.54	{8519., 8797.}	3.40255	0.0150718	Reff	0.987375	0.933985	{0.930837, 0.937193}	5.40725	ar	91 184.	76 069.	{75 683., 76 427.}	16.5763
14	R0	{1.75297, 4.45354, 0.1}	{1.75652, 4.29223, 1.49}	{{1.71582, 3.97874, 1.}, {1.79887, 4.61852, 2.}}	{2.36385, 7.25243, 1390.}	inf	10 727.	10 528.5	{9462., 11 618.3}	8.00264	0.0354849	newly	15 310.	15 198.5	{14 614., 15 712.}	2.19027	0.00959894	Reff	0.926059	0.931064	{0.920281, 0.942223}	0.856765	ar	71 263.	71 167.8	{69 062., 73 279.}	2.4748
15	R0	{1.99928, 7.1501, 0.1}	{2.13464, 8.31363, 0.05}	{{2.13505, 8.31782, 0.}, {2.13505, 8.31782, 0.}}	{6.77034, 16.273, 140.}	inf	13 996.	16 663.5	{16 466.6, 16 853.6}	19.0588	0.0757438	newly	12 056.	12 553.	{12 389., 12 707.}	4.12243	0.0175217	Reff	0.80563	0.803232	{0.799182, 0.807527}	0.412712	ar	79 602.	83 044.2	{82 734., 83 303.}	4.32423
16	R0	{2.67398, 10.3134, 1.}	{2.57012, 9.76148, 0.99}	{{2.5684, 9.75224, 1.}, {2.5684, 9.75224, 1.}}	{3.93372, 5.42194, 1.}	inf	23 578.	22 343.1	{22 153.4, 22 474.2}	5.3324	0.0237956	newly	14 011.	14 055.8	{13 916., 14 185.}	0.679609	0.0029413	Reff	0.705449	0.709747	{0.705581, 0.714155}	0.668654	ar	91 289.	90 156.8	{89 931., 90 347.}	1.25049
17	R0	{2.44634, 16.3226, 0.1}	{1.92538, 10.6367, 3.}	{{1.92538, 10.6367, 3.}, {1.92538, 10.6367, 3.}}	{21.2955, 34.8343, 2900.}	inf	22 666.	13 117.5	{12 956.4, 13 278.9}	42.1269	0.237541	newly	9419.	8218.27	{8095., 8341.}	12.748	0.0592548	Reff	0.907103	0.897776	{0.894513, 0.900784}	1.02824	ar	88 710.	77 021.9	{76 682., 77 404.}	13.1756
18	R0	{2.02168, 10.1676, 0.1}	{1.95791, 9.82151, 1.}	{{1.95791, 9.82151, 1.}, {1.95791, 9.82151, 1.}}	{3.15409, 3.40384, 900.}	inf	15 535.	14 348.3	{14 223.3, 14 480.}	7.63898	0.0345243	newly	10 392.	9955.13	{9826., 10 075.}	4.20391	0.0186773	Reff	0.996001	0.987821	{0.985228, 0.990242}	0.821279	ar	80 299.	78 314.9	{78 001., 78 633.}	2.47091
19	R0	{2.89969, 15.009, 0.1}	{2.52368, 12.5037, 0.}	{{2.52368, 12.5037, 0.}, {2.52368, 12.5037, 0.}}	{12.9673, 16.6919, 100.}	inf	28 996.	23 119.	{22 934., 23 276.2}	20.2683	0.098375	newly	12 862.	12 325.1	{12 200., 12 446.}	4.17431	0.0185305	Reff	0.881738	0.915716	{0.91171, 0.919745}	3.85356	ar	93 349.	89 398.3	{89 182., 89 589.}	4.23218
20	R0	{2.68933, 7.34928, 0.1}	{2.31954, 5.66968, 1.}	{{2.31954, 5.66968, 1.}, {2.31954, 5.66968, 1.}}	{13.7504, 22.8539, 900.}	inf	24 920.	19 458.5	{19 186.1, 19 712.}	21.916	0.107462	newly	20 039.	19 745.2	{19 488., 19 971.}	1.51974	0.00666641	Reff	0.765733	0.787123	{0.781171, 0.793409}	2.79338	ar	91 574.	86 571.9	{86 307., 86 806.}	5.46238
21	R0	{2.15352, 9.28074, 0.1}	{2.10405, 8.58066, 3.}	{{2.10405, 8.58066, 3.}, {2.10405, 8.58066, 3.}}	{2.29732, 7.54334, 2900.}	inf	17 876.	16 975.6	{16 812.6, 17 155.6}	5.03719	0.0224596	newly	12 361.	12 800.3	{12 659., 12 992.}	3.55368	0.0151441	Reff	0.8829	0.881563	{0.877715, 0.884822}	0.278881	ar	83 845.	82 428.7	{82 137., 82 713.}	1.68918
22	R0	{2.21953, 9.04387, 0.1}	{2.32069, 9.73857, 1.86}	{{2.33405, 9.83948, 2.}, {2.33405, 9.83948, 2.}}	{5.09083, 8.80541, 1774.}	inf	18 399.	20 117.1	{20 094., 20 495.2}	10.2285	0.0423881	newly	13 126.	13 300.7	{13 141., 13 434.}	1.36028	0.00585383	Reff	0.851523	0.85006	{0.844892, 0.854945}	0.380594	ar	84 856.	86 598.7	{86 505., 87 073.}	2.33246
23	R0	{2.21987, 14.5347, 1.}	{2.11132, 12.644, 0.}	{{2.11132, 12.644, 0.}, {2.11132, 12.644, 0.}}	{4.88971, 13.0079, 100.}	inf	18 816.	16 631.8	{16 479.4, 16 768.1}	11.6085	0.0535999	newly	8717.	8764.07	{8653., 8857.}	0.954342	0.00412897	Reff	0.868968	0.872595	{0.869655, 0.875754}	0.431955	ar	85 067.	82 342.	{82 131., 82 574.}	3.20337
24	R0	{2.84268, 17.9659, 1.}	{1.73798, 7.10374, 3.6}	{{1.7356, 7.10024, 3.}, {1.739, 7.10524, 5.}}	{38.8613, 60.4599, 260.}	inf	27 580.	10 050.9	{9797.34, 10 226.1}	63.5574	0.438449	newly	10 377.	9084.57	{8880., 9260.}	12.4548	0.0578218	Reff	0.920602	0.867496	{0.863968, 0.871278}	5.76866	ar	92 836.	70 704.2	{70 317., 71 045.}	23.8397
25	R0	{2.52706, 10.3679, 0.1}	{2.06638, 6.99732, 4.8}	{{2.05986, 6.93093, 5.}, {2.05986, 6.93093, 5.}}	{18.2299, 32.5098, 4700.}	inf	22 248.	14 935.3	{14 621.9, 15 077.2}	32.8691	0.173263	newly	13 481.	12 931.3	{12 668., 13 116.}	4.07729	0.0181128	Reff	0.761075	0.773319	{0.768814, 0.777683}	1.60885	ar	89 623.	81 339.5	{80 884., 81 552.}	9.24261
26	R0	{2.62835, 19.1088, 1.}	{1.85403, 9.9127, 3.}	{{1.85376, 9.90957, 3.}, {1.85376, 9.90957, 3.}}	{29.4604, 48.125, 200.}	inf	24 689.	12 292.9	{12 161.7, 12 419.7}	50.2089	0.302871	newly	8698.	8209.16	{8083., 8325.}	5.62014	0.0251514	Reff	0.928833	0.885019	{0.882543, 0.887315}	4.71709	ar	90 908.	75 118.7	{74 740., 75 521.}	17.3684
27	R0	{2.76831, 19.4155, 2.}	{2.04871, 11.6322, 3.}	{{2.04871, 11.6322, 3.}, {2.04871, 11.6322, 3.}}	{25.9944, 40.088, 50.}	inf	25 708.	14 723.1	{14 592.3, 14 836.8}	42.7295	0.242078	newly	8776.	8191.12	{8097., 8297.}	6.66454	0.0299789	Reff	0.75287	0.782214	{0.77966, 0.78484}	3.89766	ar	92 175.	80 922.8	{80 598., 81 255.}	12.2074
28	R0	{2.96609, 16.8903, 1.}	{2.00181, 8.64912, 4.86}	{{2.00488, 8.68514, 5.}, {2.00488, 8.68514, 5.}}	{32.5102, 48.7924, 386.}	inf	28 896.	14 116.3	{13 887.1, 14 314.1}	51.1478	0.311166	newly	11 093.	10 306.	{10 159., 10 438.}	7.09501	0.0319817	Reff	0.743925	0.797916	{0.794646, 0.801738}	7.25762	ar	94 024.	79 692.1	{79 293., 80 033.}	15.2428
29	R0	{2.17046, 14.39, 0.1}	{1.70777, 8.49961, 3.}	{{1.70777, 8.49961, 3.}, {1.70777, 8.49961, 3.}}	{21.3174, 40.9339, 2900.}	inf	18 683.	9756.38	{9592.93, 9912.62}	47.7794	0.282195	newly	8778.	7584.44	{7443., 7711.}	13.5972	0.0635183	Reff	0.946993	0.897566	{0.894787, 0.900327}	5.21942	ar	84 001.	69 447.7	{69 044., 69 873.}	17.3252
30	R0	{2.88772, 12.4647, 1.}	{1.9397, 5.98506, 4.15}	{{1.90341, 5.70177, 4.}, {2.05986, 6.93093, 5.}}	{32.8294, 51.984, 315.}	inf	28 167.	13 393.2	{12 454.3, 15 861.8}	52.4507	0.325229	newly	14 527.	13 733.6	{13 358., 14 247.}	5.50162	0.024702	Reff	0.836804	0.847564	{0.842329, 0.853087}	1.28596	ar	93 273.	77 656.6	{76 284., 81 322.}	16.7427
31	R0	{2.46831, 8.14308, 0.1}	{2.17679, 6.63903, 0.51}	{{2.14326, 6.48739, 0.}, {2.25976, 7.03829, 2.}}	{12.7157, 19.8801, 554.}	inf	22 336.	17 953.9	{17 049., 19 411.7}	21.1496	0.102971	newly	17 282.	16 784.5	{16 479., 17 257.}	3.06776	0.0135666	Reff	0.883087	0.871589	{0.865517, 0.877031}	1.30204	ar	88 762.	83 730.6	{82 866., 85 629.}	5.9541
32	R0	{1.93236, 7.38987, 0.1}	{2.6536, 13.2503, 3.}	{{2.6536, 13.2503, 3.}, {2.6536, 13.2503, 3.}}	{37.3245, 79.304, 2900.}	inf	13 853.	24 974.1	{24 808.1, 25 126.}	80.2792	0.25594	newly	12 032.	12 602.5	{12 471., 12 734.}	4.74161	0.020107	Reff	0.884615	0.919684	{0.91517, 0.924008}	3.96435	ar	77 654.	90 906.5	{90 759., 91 060.}	17.0661
33	R0	{2.82251, 17.6954, 1.}	{2.31035, 12.8066, 3.}	{{2.31035, 12.8066, 3.}, {2.31035, 12.8066, 3.}}	{18.1455, 27.6276, 200.}	inf	27 972.	20 389.7	{20 237.6, 20 543.9}	27.1069	0.137321	newly	10 722.	10 886.1	{10 760., 10 996.}	1.55223	0.00667433	Reff	1.00304	0.996208	{0.992449, 0.999635}	0.681054	ar	92 745.	86 396.5	{86 154., 86 606.}	6.84511
34	R0	{1.87083, 12.3994, 1.}	{2.39393, 19.7817, 3.}	{{2.39393, 19.7817, 3.}, {2.39393, 19.7817, 3.}}	{27.9606, 59.5373, 200.}	inf	12 842.	22 365.7	{22 209.1, 22 514.4}	74.1608	0.240945	newly	7135.	7916.65	{7821., 8026.}	10.9552	0.0451239	Reff	1.01449	1.07016	{1.06657, 1.07368}	5.48727	ar	76 282.	88 025.4	{87 868., 88 240.}	15.3948
35	R0	{2.95779, 16.3003, 1.}	{2.42785, 11.9984, 0.19}	{{2.44101, 12.0941, 0.}, {2.44101, 12.0941, 1.}}	{17.9167, 26.3917, 93.}	inf	29 124.	22 279.	{22 269.4, 22 771.4}	23.5031	0.117411	newly	12 158.	12 626.5	{12 490., 12 769.}	3.99071	0.0169845	Reff	1.01659	0.999526	{0.99443, 1.00538}	1.67875	ar	93 873.	88 169.3	{88 187., 88 719.}	6.07596
36	R0	{2.18718, 14.261, 3.}	{2.2143, 14.4309, 4.}	{{2.2143, 14.4309, 4.}, {2.2143, 14.4309, 4.}}	{1.24015, 1.19125, 33.3333}	inf	18 672.	20 140.3	{19 994.3, 20 266.5}	7.86358	0.0328693	newly	8858.	9380.71	{9262., 9489.}	5.90099	0.0248781	Reff	0.915641	0.894484	{0.891684, 0.897643}	2.31062	ar	84 379.	85 364.1	{85 071., 85 637.}	1.16747
37	R0	{2.44912, 9.31118, 0.1}	{2.49568, 9.77462, 1.38}	{{2.42049, 9.29056, 1.}, {2.49869, 9.80052, 4.}}	{2.58292, 5.68506, 1282.}	inf	21 427.	22 110.4	{21 213.9, 22 351.8}	4.2496	0.0183014	newly	14 344.	14 249.8	{14 131., 14 402.}	0.894381	0.00392773	Reff	0.796601	0.793651	{0.787721, 0.799105}	0.528528	ar	88 442.	89 075.1	{88 279., 89 380.}	1.01032
38	R0	{2.05949, 5.72514, 0.1}	{1.88643, 4.56746, 0.}	{{1.88643, 4.56746, 0.}, {1.88643, 4.56746, 0.}}	{8.40328, 20.2209, 100.}	inf	16 011.	12 329.9	{12 050.9, 12 607.3}	22.9909	0.113526	newly	16 782.	15 638.4	{15 302., 15 884.}	6.81468	0.0306976	Reff	0.827297	0.827218	{0.821866, 0.832114}	0.415896	ar	81 587.	76 181.4	{75 835., 76 573.}	6.62553
39	R0	{1.75505, 8.52404, 0.1}	{1.68297, 7.5059, 2.}	{{1.68297, 7.5059, 2.}, {1.68297, 7.5059, 2.}}	{4.10712, 11.9443, 1900.}	inf	10 952.	9623.45	{9436.46, 9816.85}	12.1306	0.0562124	newly	8590.	8549.97	{8342., 8726.}	1.39569	0.00609238	Reff	0.957748	0.940772	{0.937371, 0.943857}	1.77257	ar	70 925.	68 464.3	{67 958., 68 899.}	3.46945
40	R0	{2.24724, 13.7861, 1.}	{1.88096, 9.64595, 5.}	{{1.88096, 9.64595, 5.}, {1.88096, 9.64595, 5.}}	{16.2992, 30.0313, 400.}	inf	18 516.	12 278.	{12 141.1, 12 411.2}	33.6897	0.178434	newly	8935.	8230.31	{8105., 8354.}	7.88685	0.0357056	Reff	0.839861	0.820304	{0.817735, 0.822841}	2.3286	ar	85 257.	76 099.6	{75 819., 76 372.}	10.7409
41	R0	{1.77635, 8.62406, 0.1}	{1.88096, 9.64595, 5.}	{{1.88096, 9.64595, 5.}, {1.88096, 9.64595, 5.}}	{5.88891, 11.8493, 4900.}	inf	11 281.	13 049.7	{12 897.4, 13 215.2}	15.6783	0.063231	newly	8585.	8959.33	{8800., 9104.}	4.36028	0.0185048	Reff	0.87725	0.895643	{0.892427, 0.898485}	2.09663	ar	72 941.	76 174.	{75 799., 76 605.}	4.43238
42	R0	{1.80397, 5.4932, 1.}	{1.81768, 5.68021, 1.}	{{1.81768, 5.68021, 1.}, {1.81768, 5.68021, 1.}}	{0.760022, 3.40431, 0.}	inf	10 692.	11 103.6	{10 911.2, 11 341.}	3.85003	0.0163597	newly	11 817.	11 916.1	{11 690., 12 143.}	1.33291	0.00574387	Reff	0.8332	0.83205	{0.827734, 0.835509}	0.303281	ar	73 232.	73 773.3	{73 373., 74 165.}	0.749386
43	R0	{2.83096, 16.7077, 0.1}	{1.95336, 8.88243, 1.98}	{{1.95355, 8.88568, 2.}, {1.95355, 8.88568, 2.}}	{31.0002, 46.8363, 1882.}	inf	27 789.	14 269.7	{14 069.9, 14 447.3}	48.65	0.28948	newly	11 209.	10 578.5	{10 420., 10 739.}	5.62521	0.025176	Reff	0.932207	0.907302	{0.903656, 0.910844}	2.67157	ar	92 740.	78 362.5	{78 068., 78 678.}	15.503
44	R0	{2.73678, 11.6021, 1.}	{2.78925, 11.8372, 1.05}	{{2.8186, 12.0293, 1.}, {2.8186, 12.0293, 1.}}	{3.76327, 4.96857, 5.}	inf	23 763.	23 914.3	{24 136.6, 24 445.2}	3.71945	0.0173518	newly	12 451.	12 487.7	{12 365., 12 660.}	0.93366	0.00405439	Reff	0.638655	0.644484	{0.638493, 0.646929}	0.948139	ar	92 003.	92 174.7	{92 370., 92 682.}	0.942263
45	R0	{2.14235, 6.98601, 0.1}	{2.56562, 9.73364, 1.02}	{{2.5684, 9.75224, 1.}, {2.5684, 9.75224, 1.}}	{19.7571, 39.3305, 920.}	inf	15 339.	21 826.5	{21 687.5, 22 052.3}	42.2942	0.153101	newly	13 010.	13 711.4	{13 567., 13 833.}	5.39108	0.02279	Reff	0.718266	0.693622	{0.688639, 0.697936}	3.43102	ar	82 974.	90 019.1	{89 862., 90 241.}	8.49076
46	R0	{2.23409, 9.21289, 0.1}	{2.18287, 8.72564, 2.7}	{{1.84836, 6.13642, 2.}, {2.36298, 10.1198, 4.}}	{9.79314, 18.0864, 2600.}	inf	19 256.	18 416.5	{12 384.2, 21 770.2}	20.3228	0.0987931	newly	14 108.	13 938.2	{13 063., 14 470.}	3.4652	0.0153595	Reff	0.983357	0.968417	{0.937479, 0.988566}	1.78437	ar	85 163.	83 025.4	{74 616., 87 585.}	5.9464
47	R0	{2.12258, 8.15265, 0.1}	{2.19123, 8.72528, 1.21}	{{2.13505, 8.31782, 0.}, {2.33832, 9.79994, 4.}}	{3.23406, 7.02385, 1236.}	inf	17 054.	18 164.6	{17 040.9, 20 647.}	6.60125	0.0263022	newly	13 477.	13 405.8	{13 162., 13 743.}	1.42265	0.00620143	Reff	0.871829	0.874283	{0.869085, 0.879549}	0.417093	ar	82 690.	84 088.5	{82 753., 86 971.}	1.70285
48	R0	{2.71771, 10.4636, 0.1}	{2.40406, 8.591, 2.25}	{{2.28968, 7.89302, 2.}, {2.44219, 8.82367, 3.}}	{11.541, 17.8963, 2150.}	inf	26 340.	21 845.6	{19 977.4, 22 621.4}	17.063	0.0817716	newly	16 548.	16 488.9	{16 274., 16 689.}	0.761965	0.00332105	Reff	0.927228	0.927582	{0.921785, 0.932867}	0.399407	ar	91 716.	87 893.5	{86 046., 88 692.}	4.1677
49	R0	{2.44369, 6.66477, 0.1}	{2.20536, 5.63245, 2.96}	{{2.21112, 5.66494, 3.}, {2.21112, 5.66494, 3.}}	{9.75273, 15.4892, 2860.}	inf	18 124.	14 469.1	{14 316.5, 14 793.7}	20.166	0.0981223	newly	14 957.	13 787.	{13 545., 13 998.}	7.82296	0.0354451	Reff	0.592717	0.615342	{0.609939, 0.619918}	3.81721	ar	88 827.	84 568.	{84 400., 84 944.}	4.79469
50	R0	{2.77383, 17.9775, 0.1}	{1.82266, 8.17412, 4.}	{{1.82266, 8.17412, 4.}, {1.82266, 8.17412, 4.}}	{34.2909, 54.5314, 3900.}	inf	25 584.	10 424.3	{10 252.2, 10 570.7}	59.2546	0.389949	newly	9271.	7963.41	{7829., 8112.}	14.1041	0.0660629	Reff	0.747852	0.767717	{0.765014, 0.770744}	2.65618	ar	92 334.	74 078.	{73 701., 74 435.}	19.7717
